# Tailoring Surface Chemistry of Sugar-Derived Ordered Mesoporous Carbons towards Efficient Removal of Diclofenac from Aquatic Environments

**DOI:** 10.3390/ma13071625

**Published:** 2020-04-01

**Authors:** Rafał Olchowski, Emil Zięba, Dimitrios A. Giannakoudakis, Ioannis Anastopoulos, Ryszard Dobrowolski, Mariusz Barczak

**Affiliations:** 1 Department of Analytical Chemistry, Institute of Chemical Sciences, Faculty of Chemistry, Maria Curie-Skłodowska University in Lublin, 20-031 Lublin, Poland; rafal.olchowski@poczta.umcs.lublin.pl (R.O.); rdobrow@poczta.umcs.lublin.pl (R.D.); 2 Confocal and Electron Microscopy Laboratory, Center for Interdisciplinary Research, John Paul II Catholic University of Lublin, Konstantynów Sq. 1J, 20-708 Lublin, Poland; dzemski@gmail.com; 3 Department of Chemistry, Aristotle University of Thessaloniki, 54-124 Thessaloniki, Greece; dagchem@gmail.com; 4 Department of Chemistry, University of Cyprus, P.O. Box 20537, Nicosia CY-1678, Cyprus; anastopoulos_ioannis@windowslive.com; 5 Department of Theoretical Chemistry, Institute of Chemical Sciences, Faculty of Chemistry, Maria Curie-Skłodowska University in Lublin, 20-031 Lublin, Poland

**Keywords:** mesoporous carbon, bioresources, water remediation, adsorption, pharmaceuticals

## Abstract

Ordered mesoporous carbon (CMK-3), obtained from an abundant natural source, sugar, was thermochemically modified with dicyandiamide and thiourea as well as by classical oxidization with hydrogen peroxide to introduce specific surface groups. Thermochemical modifications resulted in carbon with almost unchanged porosity and altered surface chemistry while porosity of H_2_O_2_-treated carbon was seriously deteriorated. The obtained carbons were tested as sorbents of diclofenac, considered as one of the emerging water contaminants. Changes in porosity and surface chemistry of modified carbons resulted in significant differences with regard to the uptake of diclofenac. Dicyandiamide-modified carbon showed highest uptake of drugs, reaching 241 mg g^−1^ that is attributed to its developed microporosity as well as surface chemistry composed of basic groups facilitating electrostatic interactions with diclofenac anions. Desorption study showed that diclofenac is strongly bonded, albeit with a different degree depending on the modification of the CMK-carbon. The obtained results were compared with up-to-date literature regarding sorption of diclofenac by carbon-based sorbents.

## 1. Introduction

Ordered mesoporous carbons (OMCs) have been widely studied due to numerous advantages, including large surface areas and well defined in shape and size mesopores, as well as the possibility of further structural or chemical surface modification. OMCs can be considered as prosperous green-oriented materials for environmental remediation applications, since they can be derived from an abundant natural source, sugar/sucrose. The first fabrication protocols were based on the hard-template method (also called nanocasting) which involves replication of the presynthesized mesoporous silica scaffolds by filling its pores with organic precursor followed by carbonization and removal of sacrificial template [1,2,3]. Simultaneously, another protocol was developed where micelles of amphiphilic surfactant (usually triblock copolymer Pluronic P123) are acting as the template for the organic precursor [4]. Pyrolytic removal of the sacrificial template results in a carbon structure with mesopores. This self-assembly route is known as soft-templating. Theoretically, a plethora of organic precursors can be used, however, most frequently, phenol, resorcinol, formaldehyde, or carbohydrates are preferred. Apart from the two principal fabrication strategies mentioned above, there are some alternative ways to obtain mesoporous carbons, including mechanochemically induced self-assembly [5], sol-gel method [6], or other approaches [7]. Some of them were also developed to produce ordered porous carbon films [8].

Perspectives for potential applications of mesoporous templated carbons are broad and include, among others, removal of hazardous pollutants [9,10], catalytic processes [11,12], energy storage [13,14,15], and controlled drug delivery systems [16,17], as well as bioadsorption and biocatalysis [18,19]. The hydrophobic nature of OMCs makes them often poorly suited for removal of different species from aqueous systems due to the limited wetting of the sorbent surface resulting in restricted penetration of the pores by the molecules intended to be adsorbed [20]. In such cases, surface modification with appropriate surface functional groups is a strategy to overcome those difficulties [21]. A comparative study on adsorption of several nonsteroidal anti-inflammatory drugs by activated carbon and soft-templated OMCs revealed that the pristine and CO_2_ oxidized mesoporous carbons have significantly better adsorption performance than classic microporous activated carbon. The adsorption mechanism was explained by π electron interactions between the widely used anti-inflammatory drug ibuprofen/naproxen and carbon surface because of the possibility of forming Lewis acid-base complexes or hydrogen bonds [22]. Granular OMC was used for removal of several antibiotics, and the obtained carbons again proved to be better sorbents than classic activated carbon. The proposed adsorption mechanism was explained by complexation reactions and dispersive forces between the π–π* electrons in the graphene layers of the carbon and tetracycline aromatic rings [23]. Another interesting study on adsorption of antibiotics showed that OMC materials can provide fast adsorption kinetics and relevant adsorption uptakes. The strong adsorption of tetracycline was explained by the interaction of its O and N groups (e.g., phenol, amine) with the graphene surface [24].

To date, several modification protocols have been applied in order to manipulate the surface chemistry heterogeneity and structural features of ordered mesoporous carbons. Most of them are based on chemical oxidation achieved by using different agents, like (NH_4_)_2_S_2_O_8_, H_2_O_2_, H_3_PO_4_, or HNO_3_, which leads to the formation of oxygen functional groups (i.e., carbonyl, carboxyl, hydroxyl, phenol, and lactone ones) during the oxidation process [10,25]. Although HNO_3_ oxidation can result in the formation of N-containing functional groups, they are often unstable [26]. Another approach relies on the use of the heteroatoms-rich metal-free precursor for synthesis of OMC resulting in fabrication of mesoporous carbons with elevated content of heteroatom functionalities [26,27]. The main disadvantages of the latter method are: (1) Higher cost of the final material due to the use of precursors, which are usually more expensive than classical precursors, and (2) emissions of hazardous compounds during pyrolysis, like HCN or NH_3_ [26].

It is often reported that the introduction of surface functional groups can significantly enhance the sorption capability against targeted hazardous compounds. Nevertheless, the modification strategies beyond classical oxidation treatments are rarely reported, despite the fact that heteroatoms, like sulfur, nitrogen, or oxygen, can generate surface polarity [26]. Surface chemistry of the OMC materials is similar to that of classical activated carbons, so the methods followed for activation of classical carbon-based sorbents (e.g., microporous activated carbons) could be also utilized to modify OMCs. Among them, thermal treatment of the carbon materials previously impregnated with substances bearing desired heteroatoms is among the most efficient, however, not widely applied strategies. Such thermochemical treatment can lead to a remarkable doping of the carbon structure while keeping its mechanical and structural properties unchanged.

In this paper, we report the synthesis of ordered mesoporous carbon CMK-3 (Carbon Mesostructured by KAIST—Korea Advanced Institute of Science and Technology—Number 3) and its subsequent modification using two paths: (1) Classical low-temperature wet oxidation using H_2_O_2_ and (2) carbonization at 800 °C of dicyandiamide- and thiourea-impregnated CMK-3 carbons. The effects of the modification treatment on the resulting surface chemistry and porosity of the adsorption performances of the obtained carbons were also investigated. Diclofenac was chosen as model adsorbate since it is considered as one of the most hazardous pollutants representing a new class of emerging contaminants (pharmaceuticals). Nonsteroidal anti-inflammatory drugs like diclofenac are of huge concern due to their widespread use and intensive discards mainly from households and hospitals [28]. To the best of our knowledge, modified OMCs have not been tested yet as potential sorbents of diclofenac so this work is the first attempt to study in detail the adsorption of this drug. Another novelty of this study is that not-yet-explored postsynthesis modification of OMCs by dicyandiamide and thiourea was thoroughly investigated to verify if the adsorptive capabilities of modified carbons can be altered upon the surface chemistry and structural alterations.

## 2. Materials and Methods

### 2.1. Reagents

The following reagents were used as received: Pluronic P123 (P123, Sigma-Aldrich, Saint Louis, MO, USA), tetraethoxysilane (TEOS, 99%, ABCR GmbH, Karlsruhe, Germany), HCl (36%, POCH, Polish Chemical Reagents, Gliwice, Poland), ethanol (EtOH, 99.8%, POCH, Polish Chemical Reagents, Gliwice, Poland), NaOH (POCH, Polish Chemical Reagents, Gliwice, Poland), H_2_SO_4_ (96%, Merck KGaA, Darmstadt, Germany), sucrose (food sugar, Pfeifer & Langen GmbH & Co. KG, Cologne, Germany), H_2_O_2_ (30%, Sigma-Aldrich, Saint Louis, MO, USA), dicyandiamide (99%, Sigma-Aldrich, Saint Louis, MO, USA), thiourea (>99%, Sigma-Aldrich, Saint Louis, MO, USA), diclofenac sodium salt (DICL, >98%, Sigma-Aldrich, Saint Louis, MO, USA). The above-mentioned chemicals were used as received, without further purification. Deionized water (DW, resistivity <17.5 mV cm) was obtained from a Millipore system (Merck Millipore, Burlington, MA, USA).

### 2.2. Synthesis of the CMK-3 Carbon and its Further Modification

#### 2.2.1. Synthesis of SBA-15 Template 

Synthesis of SBA-15 (Santa Barbara Amorphous Number 15) template was conducted by the procedure described below. Five grams of the symmetric triblock copolymer P123 was dissolved in 180 mL HCl solution (2 M (mol L^−1^)) under vigorous stirring (500 rpm) at 40 °C. After complete dissolution of P123, 50 mmol of TEOS was added dropwise during 150 s. The mixture was stirred for 24 h, transferred into the oven and aged for 48 h at 100 °C (2 °C min^−1^) under static conditions. After the hydrothermal treatment, the solid material was recovered by filtration and subjected to a triple extraction by 255 mL of 0.2 M HCl in ethanol at 78 °C for 6 h. During the extraction process the suspension was stirred manually every 30 min. Finally, the received material was thoroughly washed by ethanol and water (until reaching the neutral pH of the filtrate), followed by drying at 100 °C for 24 h. Physicochemical characterization of SBA-15 template is presented in Figure S1.

#### 2.2.2. Synthesis of Pristine CMK-3

First, 7.5 g of sucrose was dissolved in 30 mL of water containing 600 µL of 98% H_2_SO_4_ under stirring at 300 rpm, followed by the addition of 6 g SBA-15. The mixture was heated at 100 °C for 6 h in an oven and then the temperature was raised to 160 °C (2 °C min^−1^) for another 6 h. The impregnation with sucrose was repeated by using a solution of sucrose in water (6 g of sucrose and 37.8 mL of water) and 385.8 µL of 98% H_2_SO_4_. Once again, the mixture was kept at 100 °C for 6 h and at 160 °C (2 °C min^−1^) for another 6 h. The obtained Si/C composite was carbonized at 900 °C for 3 h under nitrogen flow (1 L min^−1^). Heating rate was 10 °C min^−1^. SBA-15 template was removed by shaking the sample with 1M ethanol/water solution (volume ratio 1:1) of NaOH for 5 hours at 100 °C. After that, the sample was thoroughly washed with 1M HCl and water until Na concentration in filtrate was <1 mg L^−1^ (measured by F AAS (Flame Atomic Absorption Spectrometry) technique), followed by drying at 120 °C for 24 h. This unmodified sample was denoted as P-CMK.

#### 2.2.3. Wet Oxidation of CMK-3

First, 0.2 g of CMK-3 was placed into a beaker filled with 10% H_2_O_2_ (50 mL) and the mixture was heated for 4 h at 90 °C in a water bath under stirring at 300 rpm. Afterwards, the mixture was filtered and thoroughly washed with deionized water. The obtained sample was dried at 120 °C for 24 h. This oxidized sample is abbreviated as H-CMK in the text.

#### 2.2.4. Dicyandiamide- and Thiourea-Modification of CMK-3

First, 1 g of CMK-3 was added to a 120 mL aqueous solution containing 4.2 g of dicyandiamide or 16.6 g of thiourea. The carbon slurries were stirred at room temperature for 24 h. Next, the impregnated solids were recovered by filtration and dried at 80 °C for 24 h. Finally, they were carbonized at 800 °C (rate: 10 °C min^−1^) for 40 min under N_2_ flow. Sample modified by dicyandiamide is denoted as D-CMK and by thiourea as T-CMK.

### 2.3. Instrumental Characterization

The nitrogen isotherms were measured at −196 °C using a Quantachrome 1200e analyzer (Quantachrome Instruments, Boynton Beach, FL, USA) after degassing the samples at 120 °C in vacuum for 12 h. The micropore (V_mic_) and mesopore (V_mes_) total pore (V_tot_) volumes and pore size distributions (PSD) were estimated using the desorption branch of the isotherm by quenched solid density functional theory method (QSDFT) with a slit-shaped pore model applied [29], using ASiQwin 3.0 software (Quantachrome Instruments, Boynton Beach, FL, USA). The zeta potential was evaluated using a Zetasizer Nano ZS (Malvern Instruments, Malvern, UK). Suspensions were prepared by dispersing 2.0 mg of ball-milled samples in 2 mL of 1 mM (mmol L^−1^) KCl. The pH of an aqueous solution of drug or 1 mM KCl was adjusted by addition of the proper amount of 1 M HCL or 1 M NaOH solutions. The pH measurements were accomplished using pH meter CP-401 (Elmetron, Zabrze, Poland) equipped with a glass electrode after proper calibration. X-ray photoelectron spectroscopy (XPS) measurements were performed on a Multi-Chamber Analytical System (Prevac, Rogów, Poland) equipped with monochromatic K_α_-Al radiation (1486.6 eV (electron volt)) (Gammadata Scienta, Uppsala, Sweden) and an X-ray power of 450 W. Binding energies were referenced to the carbon C1s peak at 285 eV. Empyrean (PANalytical, Malvern, UK) diffractometer (CuK_α_ radiation) working with 0.02° size step and 10 s time step was used to record powder X-ray diffraction (XRD) patterns. The inspection of the surface of the CMK-3 was done by using the scanning electron microscope Carl Zeiss Ultra Plus (Carl Zeiss, Jena, Germany) equipped with an energy dispersive X-ray detector BrukerAXS (Bruker, Karlsruhe, Germany). The microscope was also equipped with secondary electron (SE) and backscattered electron (BSE) detectors. All experiments were carried out under required conditions (20-kV acceleration voltage and 5-nA probe current). Raman spectra were measured on an inVia Reflex (Renishaw, Wotton-under-Edge, UK) dispersive Raman microscope with an ion-argon laser (514 nm, 20 mW). Sodium concentration in water solutions was measured by flame atomic absorption spectrometer (F AAS) (SpectrAA 880, Varian, Australia) equipped with an air–acetylene burner. Spectral bandwidth of 0.5 nm, acetylene flow rate of 3.0 L min^−1^, and air flow rate of 13.5 L min^−1^ were conventional working parameters. F AAS measurements were performed using Na hollow cathode lamp (Varian, Australia) at wavelength 589.0 nm, 0.5-nm slit, and 5-mA lamp current.

### 2.4. Sorption of DICL

In each experiment, ~10 mg of adsorbent was added to 20 mL of an aqueous diclofenac (DICL) solution and shaken using MaxQ™ Mini 4450 orbital shaker (Waltham, MA, USA) (150 rpm) for 48 h (only in the case of kinetics measurements, contact times varied to collect kinetic curves, namely 0.5, 1, 2, 5, 12, 24, 48 h). The initial DICL concentrations used to collect isotherm data were: 25, 50, 75, 100, 150, 250, 400 mg L^−1^. The adsorbed amounts were calculated from the mass balance according to the formula, a = (c_i_ − c_j_)·V·m^−1^, where a is adsorbed amount (mg g^−1^), c_i_ is the initial DICL concentration (mg L^−1^), c_j_ is the final DICL concentration (mg L^−1^), V is the volume of the solution (L) and m is the mass of the adsorbent (g). DICL concentrations were measured using the UV-VIS spectrometer Specord 200 (Analytic Jena, Jena, Germany) at wavelength 278 nm after filtration of the solution through a 0.45-µm syringe filter. All the experiments and measurements were run at 20 °C.

## 3. Results and Discussion

**Figure 1:**
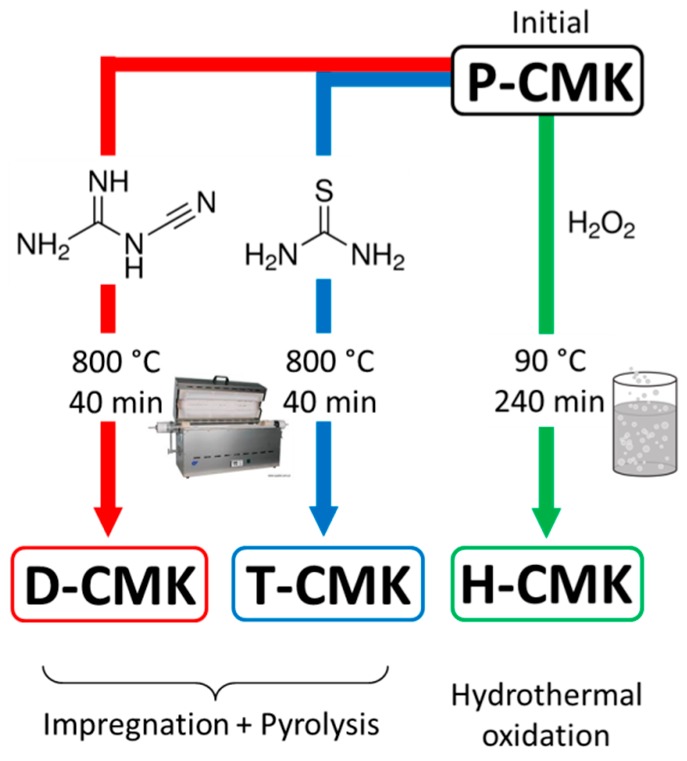
Scheme presenting three modification protocols used in this work.

The chemical modification of the initial mesoporous carbon CMK-3 was achieved by three routes, as schematically presented in Figure 1. Two of them are based on an impregnation/carbonization-based route. Initially, CMK-3 was impregnated with the N-rich precursor (dicyandiamide) or N-/S-rich one (thiourea), followed by pyrolysis under inert gas atmosphere. These modification procedures have been recently reported by us to modify activated carbon fiber textiles which are typical microporous material [30,31]. As we showed, the properties of the modified microporous textiles were significantly altered. The choice of dicyandiamide and thiourea as modifiers was made due to their rich content of nitrogen and/or sulfur; at least some fraction of those heteroatoms is expected to be incorporated in the CMK-3 framework.

The third obtained sample was received following a wet oxidation treatment with H_2_O_2_. The former two treatments were previously applied to the nanoporous carbon textiles [30,31], while the third one is a classic wet-oxidation modification process.

**Figure 2:**
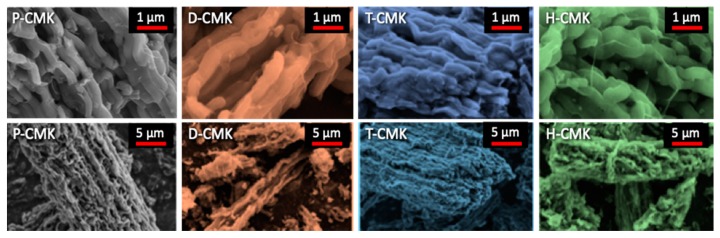
SEM microphotographs of initial and modified OMCs.

The morphology of the initial and modified OMCs was evaluated by SEM (Figure 2 and Figure S2). The images reveal a typical ‘sausage-like’ structure characteristic of OMC materials [10], i.e., micrometer-size bundles of aggregated rods. This morphology is also typical of SBA-15 template [32], which confirmed a good SBA-15 to CMK-3 replication process. The morphologies of the modified OMC materials resembled each other and also that of initial P-CMK material, which means that the morphological changes caused by modifications were negligible.

**Figure 3:**
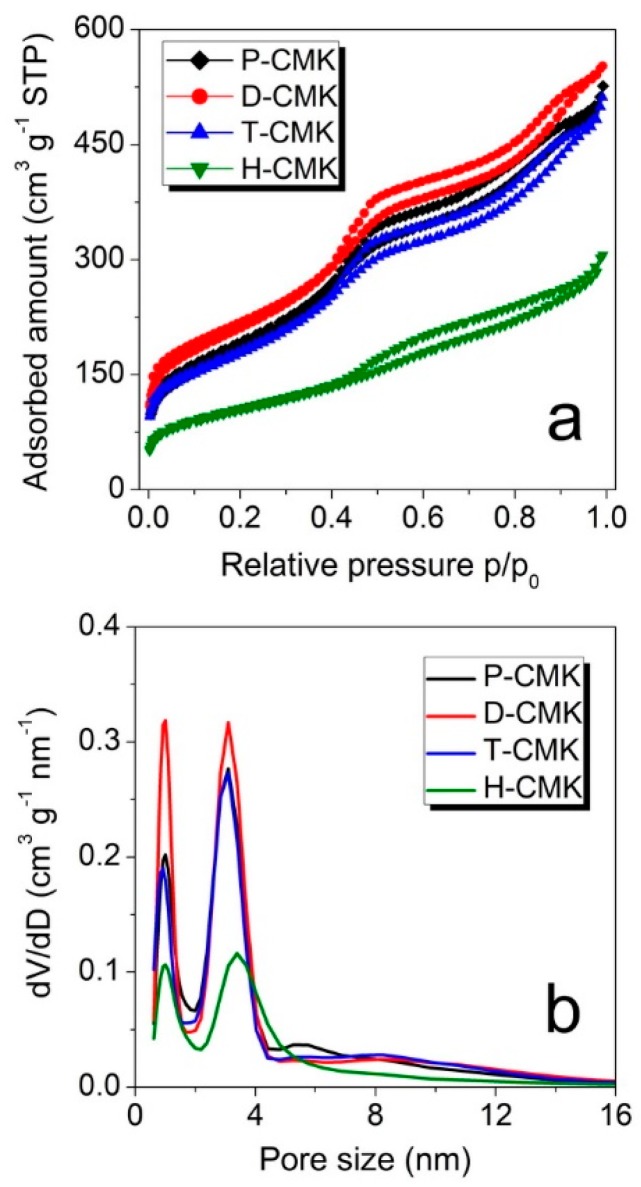
Nitrogen sorption isotherms (**a**) and corresponding pore size distributions (**b**) of the studied carbons.

The changes on the structural features were examined using nitrogen sorption measurements. The isotherm of all the samples (Figure 3a) had a typical shape for CMK-3 materials and can be classified as that of type IVa, according to the IUPAC (International Union of Pure and Applied Chemistry) classification [33]. The capillary condensation step occurred at relative pressures of about 0.40, which corresponded to the primary mesopore size of about 4 nm. The corresponding pore size distributions (Figure 3b) revealed two dominant pore fractions for all the samples with the maxima centered at ~1.0 nm and ~3.1 nm. Only in the case of the sample H-CMK this maximum was shifted towards larger pore size of ~3.4 nm, testifying to significant pore enlargement in the course of H_2_O_2_ oxidation. There were notable differences of micropore and mesopore volumes between the samples (Table 1). Dicyandiamide treatment (sample D-CMK) resulted in higher micropore and mesopore volumes when compared to the initial sample (P-CMK), while treatment with thiourea (T-CMK) did not change the porous structure significantly. On the contrary, H_2_O_2_ treatment (H-CMK) led to a drastically lowering in volume of both micro- and mesopores. The same trend was observed with relation to S_BET_ (specific surface area estimated according to Brunauer, Emmett and Teller method) values. Thus, the proposed thermochemical treatments with the use of dicyandiamide and thiourea as modification agents resulted in better preservation of porosity, while traditional wet oxidation process evidently destroyed significant parts of the porous structure.

XRD analysis (Figure S3) revealed the presence of three reflections located at 2θ angles ~1.0°, 1.7°, and 2.0°, respectively, for all the samples apart from H-CMK. These XRD signals were indexed as (10), (11), and (20) reflections, and corresponded to an ordered mesoporous structure with a 2D-hexagonal *p6mm* symmetry [34]. In the case of the H-CMK carbon, only (10) XRD reflection at 2θ angle ~1.0° was observed. This observation is in accordance with porosity results showing significant deterioration of the porous structure.

Raman spectra (Figure S4) showed two bands typically recognized for graphitized carbon materials: D band at ~1350–1358 cm^−1^ and G band at ~1591–1595 cm^−1^. The G band was assigned to the vibration of sp^2^-type carbon atoms in a hexagonal lattice, while the D band was related to the defects and disorders in structures of carbon materials. The relative intensity ratio of D to G band (I_D_/I_G_) is considered to reflect the graphitization degree of the materials [27], however, it should be interpreted with caution. The CMK-3 carbons were only slightly graphitized and should be considered as sp^2^-bonded carbons composed mainly of large polycyclic aromatic molecules rather than well-aligned graphitic domains [35]. Graphitization requires temperatures exceeding 2500 °C or sometimes lower (~1000 °C) but only with the presence of metal-based catalysts enforcing local graphitization [36,37]. It was demonstrated that the apparent G-band may result from two overlapping signals: a proper G-band but also D’ band [38]. D’ band is a disorder-induced band centered at ~1620 cm^−1^ which is frequently observed in the Raman spectra of disordered graphitic materials like graphene oxide but also for amorphous carbons [39]. The I_D_/I_G-apparent_ ratios (Table 1) indicate that the oxidation process of P-CMK carbon had little effect on the I_D_/I_G_ value. In the case of D-CMK and T-CMK carbons obtained by thermochemical modification of pristine CMK-3, the I_D_/I_G_ value increased (especially in the case of T-CMK), thus their graphitization degree was lower in comparison to P-CMK material. Surprisingly, the I_D_/I_G-apparent_ ratio of the H-CMK carbon is almost the same as in the case of the pristine P-CMK carbon, which may result from the increase of the relative share of D’ band in the apparent G band. Nevertheless, the I_D_/I_G-apparent_ values were relatively close to each other for all the carbons studied.

Additionally, the surface chemical properties of the samples were also thoroughly investigated. In Figure S5 the values of zeta potential for all the studied samples as a function of solution pH are presented. All curves intersect with pH axis at different pH values. These pH values are corresponding to the zero value of zeta potential and they are known as isoelectric points (pH_IEP_). Values of pH_IEP_ clearly differed between individual samples: P-CMK and H-CMK had pH_IEP_ values below 5 (4.5 and 3.1, respectively), most probably related to an abundance of surface oxygen groups. Obviously, after the H_2_O_2_ treatment, the number or/and character of those groups changed, which implies the noticeable shift of pH_IEP_ towards lower value. In the case of samples T-CMK and D-CMK, the value of pH_IEP_ was high (8.4 and 8.0, respectively), indicating serious changes in surface chemistry of that samples as a result of thermochemical modification with dicyandiamide or thiourea.

The values of potential zeta can be at least partially related with the protonation/deprotonation equilibria of the oxygen functional groups on the carbon surface. For example, two basic carbons (D-CMK and T-CMK) had no carboxyl surface groups (cf. Table S1—O 1s deconvolution) which were considered strongly acidic:

carbon–COOH ⟺ carbon–COO^−^ + H^+^ (3 ≤ pK_a_ ≤ 6), (1)

while they had more hydroxyl groups (cf. Table S1—O 1s deconvolution), which were weakly acidic:

carbon–OH ⟺ carbon–O^−^ + H^+^ (8 ≤ pK_a_ ≤ 11). (2)

To look more closely at the changes in surface chemistry heterogeneity as a result of the followed modifications, the superficial chemistry was inspected also by two quantitative methods: XPS and EDS (Energy-Dispersive X-Ray Spectroscopy). The first one is considered a surface-sensitive technique enabling determination of the surface composition to a depth up to several nm [40], while the second, up to several µm [41]. The first interesting conclusion was that the results collected using both techniques were nearly compatible (Table 2), albeit with two important exceptions, which will be discussed later in detail. XPS analysis reveals that the carbon content for all samples but H-CMK was higher than 91%. Highest carbon content for D-CMK carbon (93.9%) resulted most probably from deposition/incorporation of dicyandiamide fragments formed in the course of its thermal decomposition. This effect can be linked with the further development of the porous structure during dicyandiamide modification (cf. Table 1). In the case of T-CMK, the carbon content was almost unchanged, while significant decrease of carbon content to 83.7% was observed after wet oxidation using H_2_O_2_. In the same time, the oxygen content for H-CMK increased up to 15.9%, indicating the oxygen-rich surface chemistry of this sample. In contrast, thermochemical treatment with dicyandiamide and thiourea resulted in decreased oxygen content (5.2% and 4.5%, respectively) when compared with the initial P-CMK carbon (7.0%). A similar trend was observed in the case of porous carbon textiles modified with dicyandiamide and thiourea in the same way [31]. The presence of nitrogen was not evidenced by XPS for the D-CMK carbon, however, nitrogen was detected by EDS elemental analysis (1.1%). The most plausible explanation can be that the nitrogen moieties were located in the interior parts of the D-CMK structure, which was inaccessible for XPS due to the scanning depth limited to several nm. Noticeable relative differences of N and S contents by two techniques again should be attributed to the specific locations of the heteroatoms in the interior carbon matrix, thus XPS-inaccessible parts of carbon framework.

**Figure 4:**
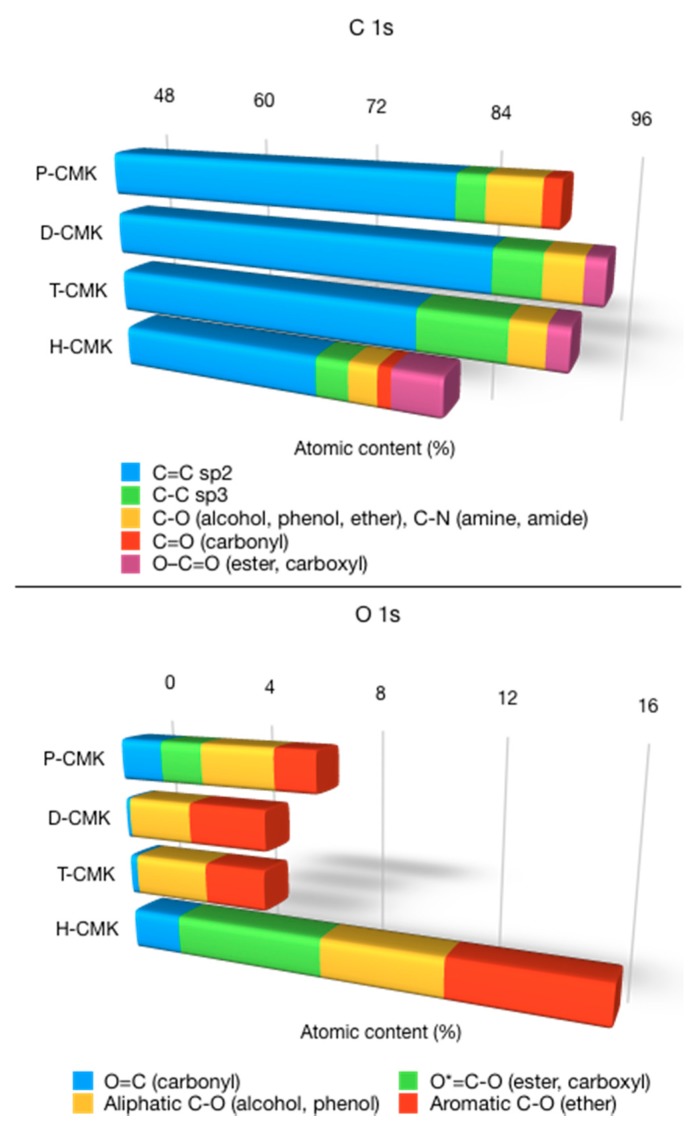
The results of the deconvolution of the XPS spectra of C 1s and O 1s core energy levels.

Deconvolution of the C1s and O1s core energy levels along with survey spectra is provided in Supplementary Materials (Figures S6–S8), while its graphical representation is shown in Figure 4. The most intensive component after deconvolution of C1s core energy level located at 283.9–284.2 eV was assigned to sp^2^-bonded (C=C) carbon structures, which were rather large polycyclic fragments than graphitic domains. The relative contribution of this signal was ~90% for P-CMK and D-CMK-3 carbons and ~85% for T-CMK and H-CMK carbons. Interestingly, T-CMK had elevated contribution of sp^3^-bonded (C–C) carbon fragments, which suggested partial cleavage of double C=C bonds, however without significant incorporation of oxygen species. The deconvolution of C1s signal shows that a wide range of different functionalities was present on the carbon surface, typical for carbon-based materials, although with different concentrations. The most striking feature of H-CMK was its high content of strongly acidic carboxyl groups (6.0%), while for the rest of the samples this content was significantly lower (0.0% for P-CMK, 1.8% for D-CMK, and 2.1% for T-CMK).

The deconvolution of O1s core energy level resulted in four signals for P-CMK and H-CMK and three signals for D-CMK and T-CMK (Figure S8). Their presence testifies to the abundance of different oxygen species on the surface, albeit with different relative shares [30]. The two most important observations coming from the analysis of deconvoluted O 1s core energy level were: (1) H-CMK exhibited significant contributions of all types of oxygen species, with the most prominent presence of strongly acidic carboxy groups, and (2) D-CMK and T-CMK had no strongly acidic carboxy groups but contained almost entirely weakly acidic phenols and basic ether groups.

In the case of T-CMK carbon, the deconvolution of N 1s and S 2p’s core energy levels resulted in the appearance of three or two signals, respectively. The most intensive component of N 1s core energy level located at 398.7 eV was assigned to pyridinic nitrogen and/or imine [30]. Two other components of N 1s core energy level located at 400.1 eV and 401.5 eV corresponded to pyrrolic nitrogen/amine and quaternary nitrogen, respectively. Both pyridinic and pyrrolic nitrogen species can be located at the edges of graphene planes and quaternary nitrogen can be located inside graphene layer. Aromatic sp^2^-hybridized nitrogen, such as in pyridine, and quaternary nitrogen led to substantial electron deficiency in the aromatic ring, which provided a positive charge on the graphene surface. On the other hand, the S 2p’s core energy level components located at 164.2 eV (S 2p_3/2_) and 165.4 eV (S 2p_1/2_) corresponded to thiols, bisulfides, and R_2_-SO groups, which can be located at the edges of graphene layers [30].

Those considerations were confirmed by observing the initial pH values (see Table 1) of the solution containing carbons immersed in water and stabilized overnight. It can be seen that the initial pH of CMK-P carbon was 4.5, indicating an abundance of acidic oxygen groups (e.g., carboxyl), which hydrolyze releasing H_3_O^+^ ions, resulting in lowered pH value. H_2_O_2_ oxidation resulted in an even more acidic surface (pH = 3.0) that suggests remarkable increase of the oxygen acidic groups on the surface, which was clearly confirmed by the deconvolution of O 1s core energy level. In contrast, the initial pH of both thermochemically modified carbons was basic (i.e., 7.8 for D-CMK and 8.3 for T-CMK), indicating basic surface functionalities. Again, this was in total agreement with the deconvoluted O 1s core energy level, where abundancies of basic ether groups were present in the case of those thermochemically treated carbons. For better clarity in Figure S9 content of functional group versus pH_IEP_ of the carbons studied was presented.

It should be noted at this point that the acidity/basicity balance of carbons was not only related to the presence of more or less acidic surface oxygen functionalities but also to the delocalized π-electrons in polyaromatic/graphene domains, which had a basic character, i.e., protons were hydrated according to C_π_ + 2H_2_O → C_π_H_3_O^+^ + OH^−^ [42]. Thus, the analyzed surface basicity was not straightforward because it was not entirely associated with surface oxygen complexes. Three general observations derived from the literature can be shortly summarized as following [42]: (1) Acidic sites were associated with the surface oxygen complexes, although some of them were less acidic (e.g., phenols) while others were more acidic (e.g., carboxyls) [43]; (2) basic sites were concentrations of delocalized π-electrons within the polyaromatic/graphene domains of the carbon structure; and (3) the increase of the nitrogen content of the polyaromatic/graphene domains enhanced remarkably the basicity. Considering all those aspects, it can be concluded that the basicity of the D-CMK and T-CMK carbons resulted from both simultaneously occurring factors: The increasing content of delocalized π-electrons and decreasing content of the acidic oxygen groups, during both thermochemical treatments, oxygen groups were stripped from the surface. Since dicyandiamide and thiourea molecules do not contain oxygen, the stripped oxygen species cannot be replaced by other oxygen species. As a result, thermochemical activation led to materials with increased carbon content and decreased oxygen content. It was demonstrated before in the literature that the surface basic sites of carbons are mainly of the Lewis type [44]. Additionally, incorporation of basic nitrogen groups also remarkably contributed to the increased basicity. In the case of H_2_O_2_ oxidation, the opposite processes occurred: large amount of acidic oxygen groups were generated which localized π-electrons, thus suppressing the effect of the π-electrons responsible for carbon basicity. XPS revealed that carbon content dropped from 91.0% to 81.8% during H_2_O_2_ oxidation with a simultaneous increase of oxygen from 7.0% to 15.9%, which clearly supported those considerations.

Well-developed porosity and rich surface chemistry make the obtained materials good candidates to be used as sorbents. Two main reasons make the obtained carbons potentially useful for drug removal: (1) a mesoporous structure providing fast transport of bulky drug molecules by diffusion through the mesopores towards the adsorption centers, and (2) tunable surface chemistry, which can be used to tailor the strength of interactions between adsorbates and the carbon surface. To verify this hypothesis and if it is applicable for pharmaceuticals’ removal, and specifically of diclofenac, adsorption experiments were conducted. On the basis of our previous studies [34,45] as well as the literature review (see Table S2), the pH value was set as 5.5–6.0. Around pH ≈ 4, the formation of not-dissociated acidic and minimally soluble form of diclofenac took place (pK_a_ value of DICL was 4.15 [34]) which made the interpretation of the adsorption results erroneous due to DICL precipitation. However, at pH > 5.5, the soluble ionized form of DICL was almost exclusively present in the solution, which made it possible to run adsorption experiments using unbuffered solutions of pH ≈ 5.5.

**Figure 5:**
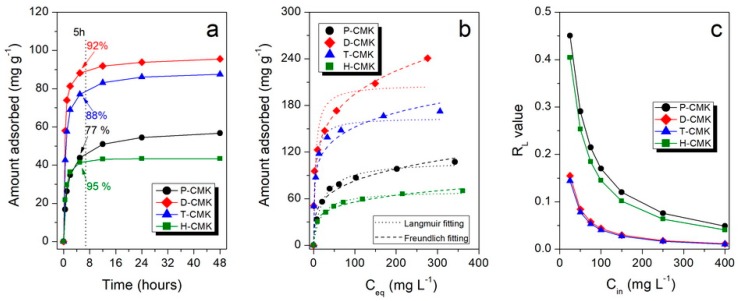
(**a**) DICL adsorption kinetics onto the studied CMK materials (initial concentration of DICL: 50 mg L^−1^), (**b**) adsorption isotherms along with their fitting using Langmuir and Freundlich models, (**c**) values of separation factor (R_L_) for different DICL initial concentrations used in this study.

Figure 5a collects the adsorption kinetics of DICL for the herein studied samples. As shown, the adsorption kinetics were relatively fast for carbon materials, mainly due to the open, interconnected three-dimensional pore structures replicated from the SBA-15 template. After 5 h, all the samples but P-CMK had uptakes close to equilibrium values (92%, 88%, and 95%, for D-CMK, T-CMK, and H-CMK, respectively). In the case of initial P-CMK carbon, only 77% of equilibrium value was adsorbed after that time. This behavior of P-CMK sample can be explained by its low wettability due to few oxygen groups on the surface. Although the total oxygen content was high (7.0% by XPS), most of those groups were located in the micropores but not in larger trafficking pores; thus, the migration of the DICL ions to the adsorption was slowed down. It is worth noting that the porous structure of the sample H-CMK was predominantly mesoporous; thus, the adsorption process was fast and was additionally facilitated by the abundance of surface oxygen groups introduced in the course of H_2_O_2_ oxidation. In the case of classical microporous activated carbon, Norit SX2 was used for comparative purposes, and the adsorption was significantly slower and the equilibrium was reached after 8 days (basic characterization of Norit SX2 and its adsorption performance is presented in Figures S10 and S11). This was due to the closed and tortuous microporous structure without a significant fraction of larger pores, which would act as transporting/diffusion channels for DICL delivering to adsorption centers located in micropores.

Adsorption isotherms, presenting the dependence of the adsorbed amount of DICL on its equilibrium concentration, are shown in Figure 5b. The highest sorption capacities (SCs) were observed in thermochemically modified samples, D-CMK and T-CMK (241 and 172 mg g^−1^, respectively), while for the initial P-CMK and H_2_O_2_-treated H-CMK samples those values were significantly lower (107 and 70 mg g^−1^, respectively). Another interesting observation is that D-CMK and T-CMK carbons can adsorb as much as 99% and 98% of DICL, respectively, from the solution with its lowest concentration tested (i.e., 25 mg L^−1^). This means that almost quantitative adsorption of DICL was provided by those materials, while for P-CMK and H-CMK, corresponding uptakes were much lower: 67% and 61%, respectively. The key role of positive influence on adsorptive capability upon surface chemistry modification can be derived by the comparison of the sorption capacities per S_BET_ (Table 3). All modifications led to an increase of the surface uptake capability, although the positive impact compared to P-CMK was the lowest for H-CMK (+16%) and the highest for D-CMK (+ ~100%).

The sorption equilibrium data were modeled with Langmuir and Freundlich adsorption models (the appropriate equations can be found in our previous works [46,47]), and the fitted results are presented in Figure 5b while the fitting parameters and determination coefficients, R^2^, in Table 3. The fitting clearly shows that for all the samples, except D-CMK, the Langmuir models assuming the formation of adsorbed monolayer provides fit better, and that is reflected in R^2^ values. Interestingly, only in the case of the D-CMK was the Freundlich model significantly better (what can be easily seen by comparing the fitted Freundlich and Langmuir curves).

Explanation of the above-mentioned phenomenon is strictly linked to the mechanisms governing the adsorption of DICL. Diclofenac has been reported to interact with carbonaceous surfaces via three types of interactions: (1) van der Waals electrostatic forces, (2) hydrogen-bonding formation, and (3) noncovalent π-π stacking interactions between the aromatic rings. In the case of the sample D-CMK nonspecific electrostatic interaction between the surface and DICL anions was particularly strong because of the positively charged surface (supported by the positive value of ζ potential). In contrast, T-CMK surface charge was almost neutral; thus, electrostatic interactions were not the favorable driving force for DICL adsorption. Most probably, specific interactions between DICL anion and N- and S- surface groups were partially responsible for high DICL uptake, although it cannot be fully supported on the basis of the collected data.

In Table 2, the pH changes of DICL solution before and after the adsorption process are collected. In the case of both thermochemically modified carbons, the change of solution pH towards higher values can be explained by the participation of H_3_O^+^ cations from the solution into the formation of hydrogen bonds between DICL anions and carbon surface. Thus, the solution pH after DICL adsorption was at high level. An even stronger effect was observed in the case of P-CMK and H-CMK-3 carbons (increase of pH by 2.5 units).

The lowest uptakes of DICL onto P-CMK and H-CMK can be related to the strong electrostatic repulsion between the negatively charged surface and DICL anions, as well as to a preferable monolayered adsorption via the carboxylic groups that prevent a second adsorption layer, as above mentioned. In the case of the H-CMK, surface area (375 m^2^ g^−1^) and volume of micropores (0.097 cm^3^ g^−1^) were the lowest among the samples studied, and so this can be the reason behind the lowest DICL uptake (only 70 mg g^−1^) compared to the rest of the samples. Clearly, the type of functional groups present on the surface and porous structure were the two most important and intercorrelated parameters affecting the sorption efficiency. Well-developed micro- and mesoporous structure without proper functionalization cannot provide fast and high adsorption, which was clearly seen for the P-CMK sample: Lack of polar surface slowed down the adsorption kinetics due to low wettability, but also resulted in insufficiently strong interaction of DICL with the adsorption centers.

Knowing the value of the Langmuir equilibrium constant (related to the adsorption energy), K_L_, allows us to calculate so-called separation factor, R_L_, which is a dimensionless constant accounting for affinity between the sorbate and sorbent [34]. In general, R_L_ = 1 corresponds to the linear adsorption isotherm, when R_L_ << 1 the adsorption is highly favorable (in the extreme but theoretical case where R_L_ = 0, adsorption is considered irreversible). R_L_ values for different initial concentrations are presented in Figure 5c and for the highest initial concentration used (400 mg L^−1^) also in Table 3. In the case of D-CMK and T-CMK, the R_L_ values are lower than the corresponding values of P-CMK and H-CMK for any initial concentration, indicating high adsorption process.

To describe in more detail the interactions between DICL and the studied materials, desorption tests were run. A 0.9 wt. % solution of NaCl was used as DICL desorption medium from the loaded samples. Its desorption efficiency was confirmed by us previously in the case of silica materials, where an increase of the ionic strength resulted in significant desorption of DICL [48,49]. The relative desorption efficiencies (RDE) are given in Table 3. It can be seen that there were remarkable differences between the samples: Low RDE was observed for D-CMK (14.3%), moderate RDE for P-CMK and T-CMK (28.5 and 38.6, respectively), and almost total desorption for H-CMK (92.4%). The observed differences can be explained by distinct alterations of surface chemistry and porosity of the carbon studied. D-CMK had the highest surface charge, as indicated by its positive ζ potential. At the same time, it had highest volume of micropores, where the bulky DICL ions can be strongly and irreversibly adsorbed. The micropores of CMK-3 carbon are textural slit-like micropores (i.e., they do not result from the templating protocol but are formed on the course of carbonization, similarly to the micropores of activated carbon). Due to the slit-like shape of the pores, even big molecules can enter the pore spaces if only one of the molecular dimensions of the adsorbate is smaller than the pore size, regardless of its two remaining dimensions. The length of diclofenac was approximately 1.25 nm (taking into account van der Waals’ radii of the outermost protons). However, its height was less than 1 nm so there were no serious steric hindrances which could limit free diffusion of diclofenac into the micropore space [34].

Low RDE of only 14.3% is most probably associated with the desorption of DICL anions adsorbed in mesopores due to the increase of ionic strength suppressing electrostatic interactions between the positively charged surface and negatively charged DICL ions. However, it is plausible to assume that DICL anions adsorbed in narrow slit-like micropores were bonded via electrostatic interactions, hydrogen bonds, and π-π stacking, which are difficult to brake due to limited water access to the filled micropores. In the case of the P-CMK and T-CMK, the attractive electrostatic interactions were weaker (particularly for P-CMK) and the volume of micropores was significantly lower than that of D-CMK. Both factors resulted in higher desorption efficiency. In the case of H-CMK, the surface was negatively charged and the volume of micropores able to provide the strong binding forces was low, so diclofenac could be desorbed almost quantitatively.

**Figure 6:**
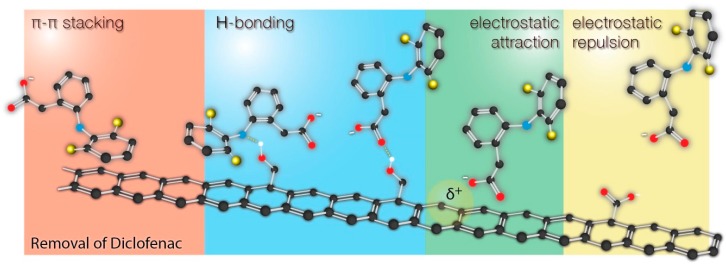
Different adsorption mechanisms involved in the sorption of diclofenac by carbon-based materials.

The collected results were compared with the current up-to-date papers reporting adsorption of diclofenac by different carbonaceous materials. As it can be seen, most of the studies indicate adsorption mechanism based on π-π interactions, hydrogen bonding, and electrostatic interactions. The possible interactions are schematically shown on Figure 6.

The possible interactions of diclofenac (as well as the other pharmaceuticals with similar structure) with the surface are shown in Figure 6. It should be clearly underlined here that it is difficult or even impossible to attribute the enhanced sorption of diclofenac to the presence of specific functional groups on the surface. The chemistry of carbon surface is very rich in various chemical groups. All modification protocols (including those described in this work) do not qualitatively transform one type of groups into another, but rather change the relative contributions of those groups to the total surface chemistry. Changes of surface chemistry result in the changes of surface charge, π-basicity (functional groups localize π electrons), which results in different adsorption properties. As a result, the changes of surface chemistry affect even more nonspecific interactions (π-π stacking and electrostatic interactions) than the specific interactions of diclofenac with particular functional groups (hydrogen bonding). Moreover, the adsorption performance was also linked with the changes of porous structure, making the discussion even more complex. For example, due to the significant fraction of microporosity, some surface groups may be accessible for H_3_O^+^ ions but not to diclofenac. All these factor make it difficult to propose one strict adsorption mechanism of diclofenac onto carbon-based materials. On the contrary, such a straightforward mechanism can be found in the case of silica-based materials with a similar mesoporous structure but a well-defined surface chemistry [34,45,50,51].

## 4. Conclusions

Different modification routes used in this study resulted in mesoporous CMK-3 carbons with various surface chemical heterogeneity and structural features. Thermochemical modification with dicyandiamide or thiourea resulted in the ordered mesoporous carbons with high basicity caused by the presence of significant amounts of heteroatom-based functional groups but also increased sp^2^-hybridized carbon content. The XPS elemental analysis revealed a remarkable decrease of the total oxygen content for thermochemically treated carbons with simultaneous increase of the total carbon content (94.8% for D-CMK and 92.4% for T-CMK). In contrast, classical wet oxidation resulted in the elevated oxygen content with a predominant fraction of acidic surface groups (mainly phenols and carboxyls). The porous structure was either intact (T-CMK) or altered (D-CMK) during modification. In contrast, classical wet H_2_O_2_ oxidation led to a material with deteriorated porosity and ordering.

The adsorption of diclofenac sodium onto modified mesoporous carbons was strongly dependent on both surface chemistry and porous structure. The dicyandiamide-modified sample (D-CMK) exhibited the highest adsorption capacity (241 mg g^−1^) and the lowest desorption extent (14.3%), while the H_2_O_2_-oxidated H-CMK sample had the lowest adsorption capacity (70 mg g^−1^) and the highest desorption (92.4%). The collected results show that the adsorption of pharmaceuticals on carbon-based materials depends on a complex interrelationship between the factors related to surface chemistry and porous structure as well as the wettability of the carbon surface.

## Figures and Tables

**Table 1:** Results derived from the nitrogen sorption, ζ potential, Raman, XPS measurements, and pH measurements.

Sample	S_BET_ (m^2^ g^−1^)	V_mic_ (cm^3^ g^−1^)	V_mes_ (cm^3^ g^−1^)	V_tot_ (cm^3^ g^−1^)	Potential ζ (mV)	I_D_/I_G_	C_sp3_/C_sp2_ Ratio	Initial pH	Final pH	pH_IEP_
P-CMK	663	0.167	0.567	0.734	−23.3	0.90	0.10	4.5	7.0	4.5
D-CMK	753	0.201	0.594	0.795	19.0	0.94	0.11	7.8	8.5	8.0
T-CMK	636	0.155	0.555	0.710	−2.4	0.98	0.17	8.3	9.2	8.4
H-CMK	375	0.097	0.321	0.418	−28.6	0.89	0.18	3.0	5.5	3.1

S_BET_, specific surface area; V_mic_, micropore volume; V_mes_, mesopore volume; V_tot_, total pore volume.

**Table 2:** Elemental analysis of the samples studied as given by EDS and XPS methods.

Sample	EDS Elemental Analysis *					XPS Elemental Analysis **				
	C wt.%	O wt.%	N wt.%	S wt.%	Cl wt.%	C wt.%	O wt.%	N wt.%	S wt.%	Cl wt.%
P-CMK	91.0	7.9	0.0	0.4	0.1	91.0	7.0	0.0	0.0	2.0
D-CMK	93.9	4.3	1.1	0.1	0.0	94.8	5.2	0.0	0.0	0.0
T-CMK	91.1	6.9	0.8	0.8	0.0	92.4	4.5	1.7	1.4	0.0
H-CMK	83.7	15.0	0.0	0.1	0.3	81.8	15.9	0.0	0.0	0.8

* EDS elemental analysis averaged from six experimental points. Elements like Na, Si, K, and Ca were also detected (less than 1% and they were not taken into consideration on the wt. % expression). ** Elements like Ca or Si were also detected (less than 1%).

**Table 3:** Sorption capacities (SC) of DICL and parameters calculated from the Langmuir and Freundlich fitting.

Sample	SC	SC/S_BET_	Langmuir Fitting				Freundlich Fitting			RDE
	(mg g^−1^)	(mg m^−2^)	q_m_	K_L_	R^2^	R_L(400)_	K_F_	n	R^2^	(%)
P-CMK	107	0.161	109	0.05	0.993	0.05	27	0.25	0.924	28.5
D-CMK	241	0.320	207	0.22	0.869	0.01	75	0.21	0.994	14.3
T-CMK	172	0.270	164	0.24	0.941	0.01	71	0.17	0.937	38.6
H-CMK	70	0.187	70	0.06	0.983	0.04	21	0.21	0.958	92.4

## Supplementary Materials

The following are available online at https://www.mdpi.com/1996-1944/13/7/1625/s1. Figure S1: Characterization of the SBA-15 template: Nitrogen sorption isotherm (a), XRD diffractogram (b), and SEM images (c,d). Figure S2: SEM microphotographs of the carbons studied. Figure S3: XRD diffractograms of the carbons studied. Figure S4: Raman spectra of the carbons studied. Figure S5: Values of zeta potential of the studied carbons as a function of pH. Figure S6: XPS survey spectra for the carbons studied: P-CMK (a), H-CMK (b), D-CMK (c), T-CMK (d). Figure S7: Deconvolution of C 1s energy level for the carbons studied: P-CMK (a), H-CMK (b), D-CMK (c), T-CMK (d). Figure S8: Deconvolution of O 1s energy level for the carbons studied: P-CMK (a), H-CMK (b), D-CMK (c), T-CMK (d). Figure S9: Functional group content versus pH_IEP_ of the carbons studied. Figure S10: Nitrogen adsorption isotherm of Norit SX2 (left), SEM images of Norit SX2 (right). Figure S11. Comparison of DICL adsorption kinetics onto the studied CMK materials and Norit SX2 carbon (initial concentration of DICL: 50 mg L^−1^). Table S1: Results of the deconvolution of the XPS C 1s and O 1s core energy levels. Table S2: Comparison of DICL maximum adsorption capacities by carbon-derived sorbents reported in the literature.
