# Supplementary material for: Tailoring Surface Chemistry of Sugar-Derived Ordered Mesoporous Carbons towards Efficient Removal of Diclofenac from Aquatic Environments
Source: Materials (Basel). 2020 Apr 1;13(7):1625. doi: 10.3390/ma13071625 (PMC7178346; doi:10.3390/ma13071625)
Supplement: Supplementary file 1 [file materials-13-01625-s001.pdf]

materials

MDPI

Article

Tailoring Surface Chemistry of Sugar-Derived Ordered Mesoporous Carbons towards Efficient Removal of Diclofenac from Aquatic Environments

Rafał Olchowski [1], Emil Zięba [2], Dimitrios A. Giannakoudakis [3], Ioannis Anastopoulos [4], Ryszard Dobrowolski [1] and Mariusz Barczak [5,*]

Received: 29 February 2020; Accepted: 28 March 2020; Published: date

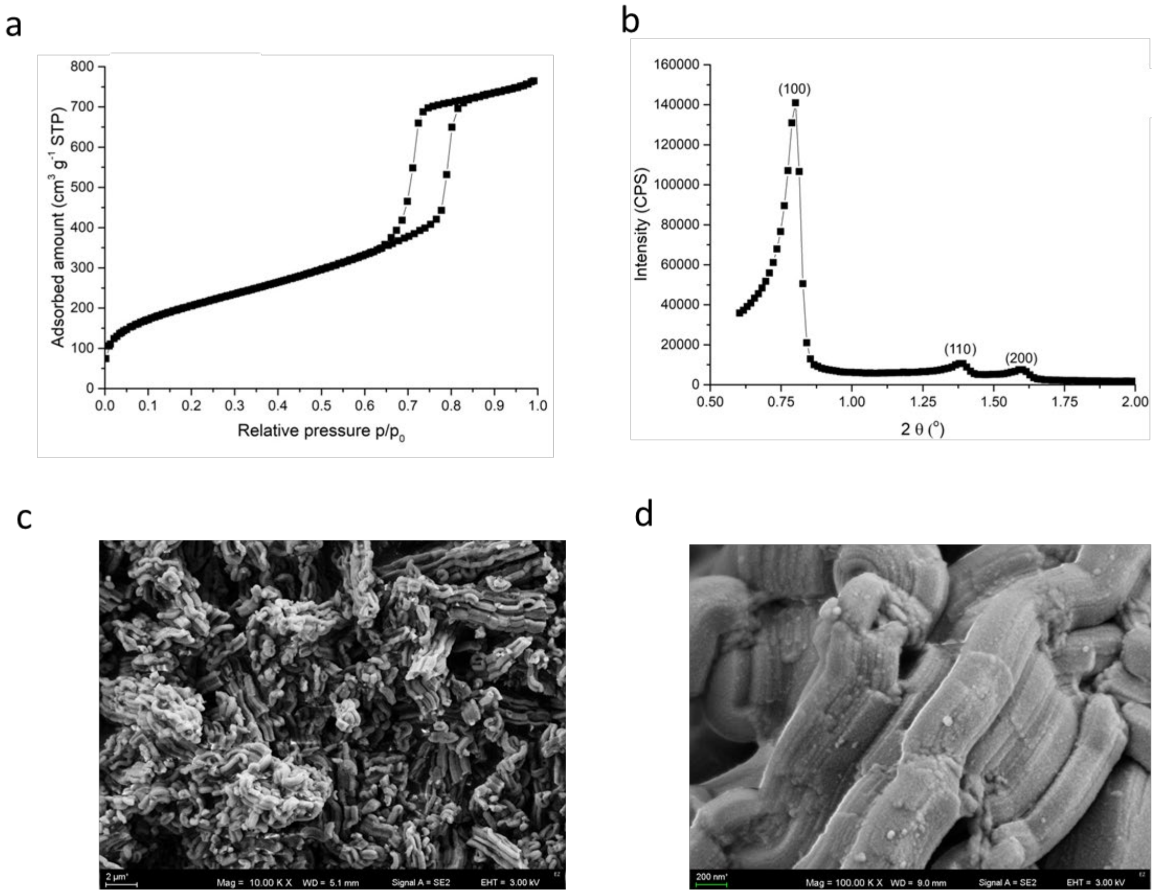

Figure S1. Characterization of the SBA-15 template: nitrogen sorption isotherm (**a**) XRD diffractogram (**b**) and SEM images (**c**,**d**).

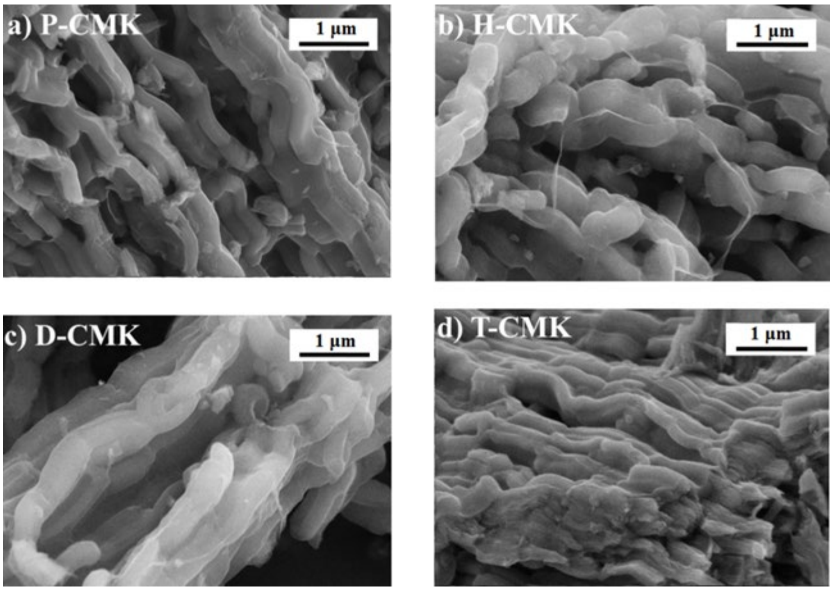

Figure S2. SEM microphotographs of the carbons studied.

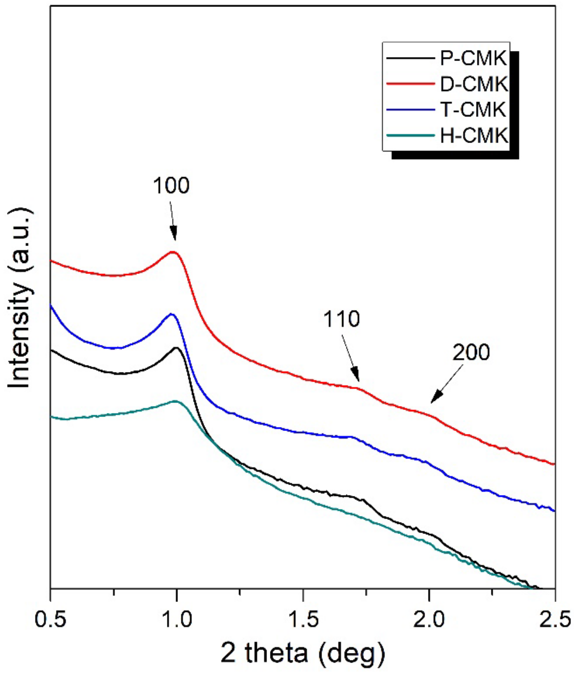

Figure S3. XRD diffractograms of the carbons studied.

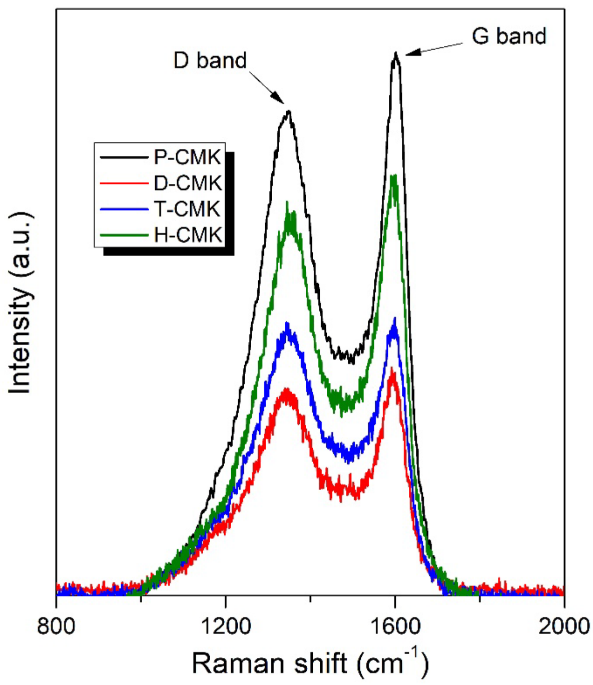

Figure S4. Raman spectra of the carbons studied.

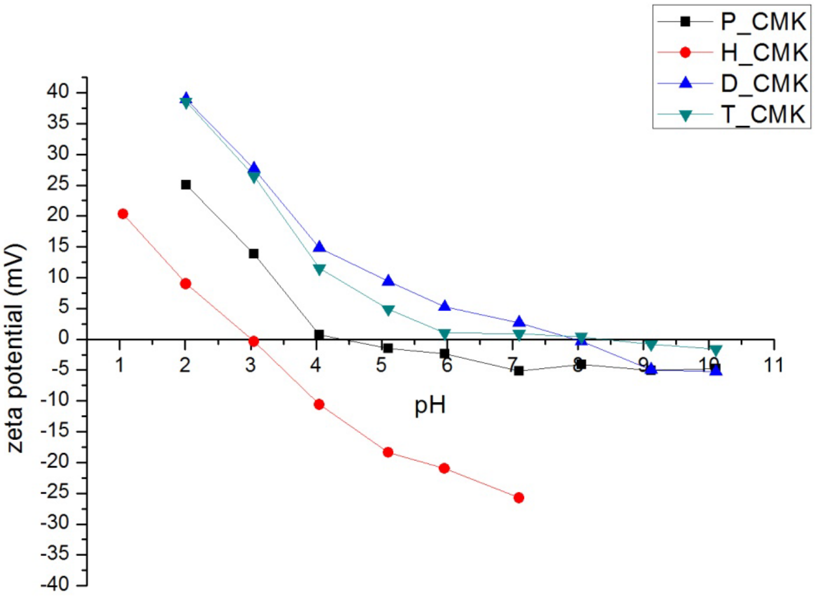

Figure S5. Values of zeta potential of the studied carbons as a function of pH.

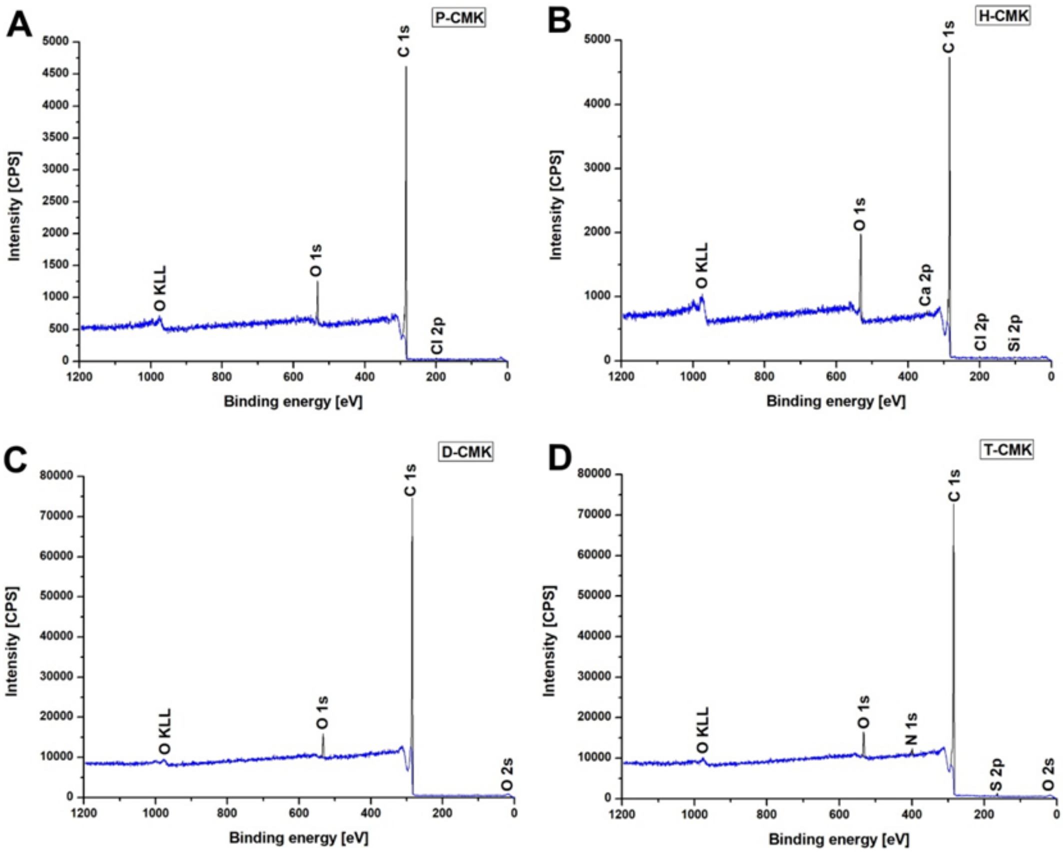

Figure S6. XPS survey spectra for the carbons studied: P-CMK (**a**), H-CMK (**b**), D-CMK (**c**), T-CMK (**d**).

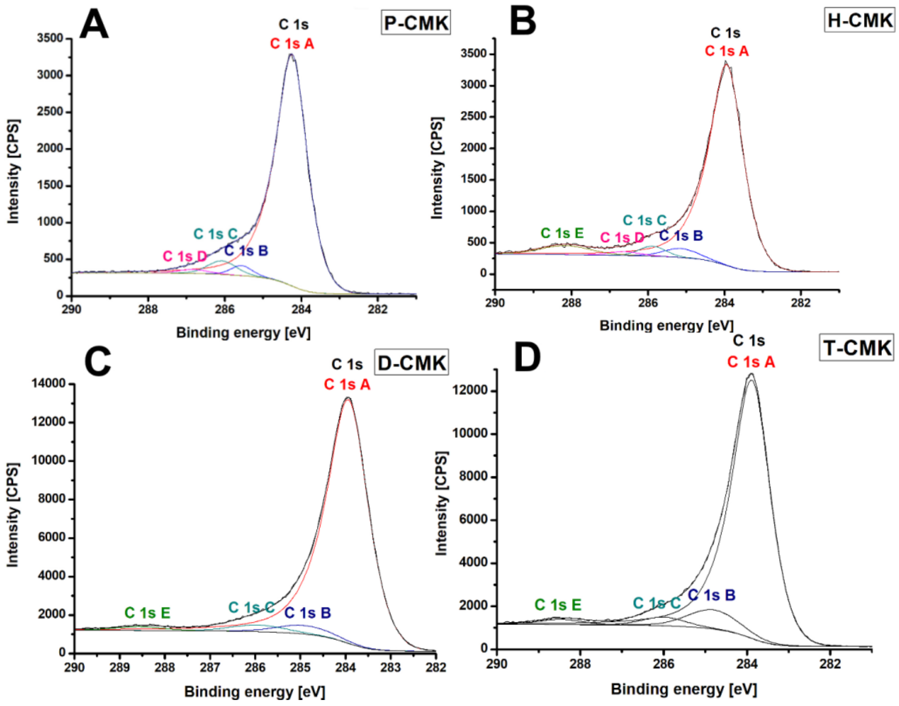

Figure S7. Deconvolution of C 1s energy level for the carbons studied: P-CMK (**a**), H-CMK (**b**), D-CMK (**c**), T-CMK (**d**).

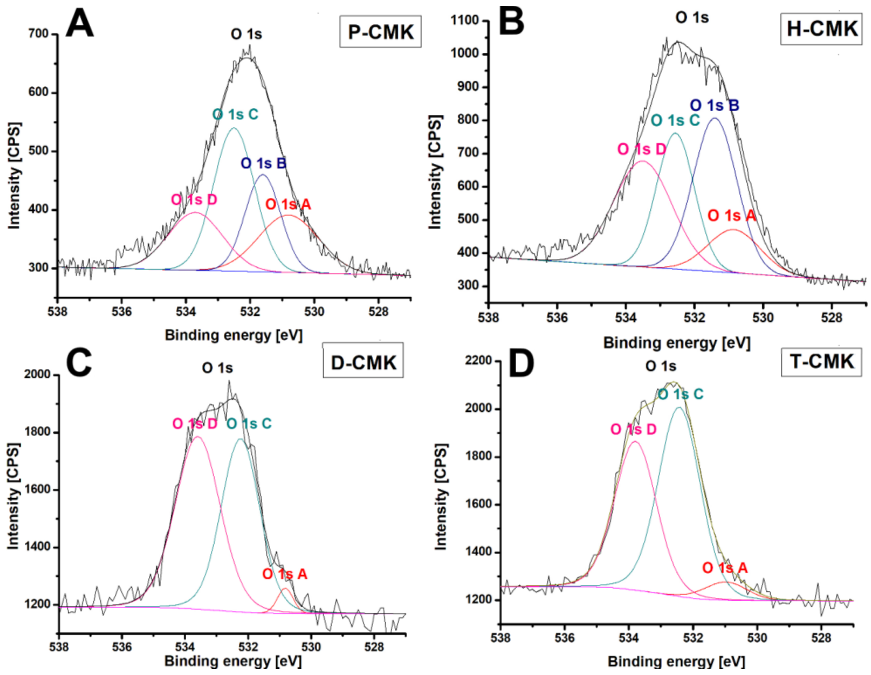

Figure S8. Deconvolution of O 1s energy level for the carbons studied:P-CMK (**a**), H-CMK (**b**), D-CMK (**c**), T-CMK (**d**).

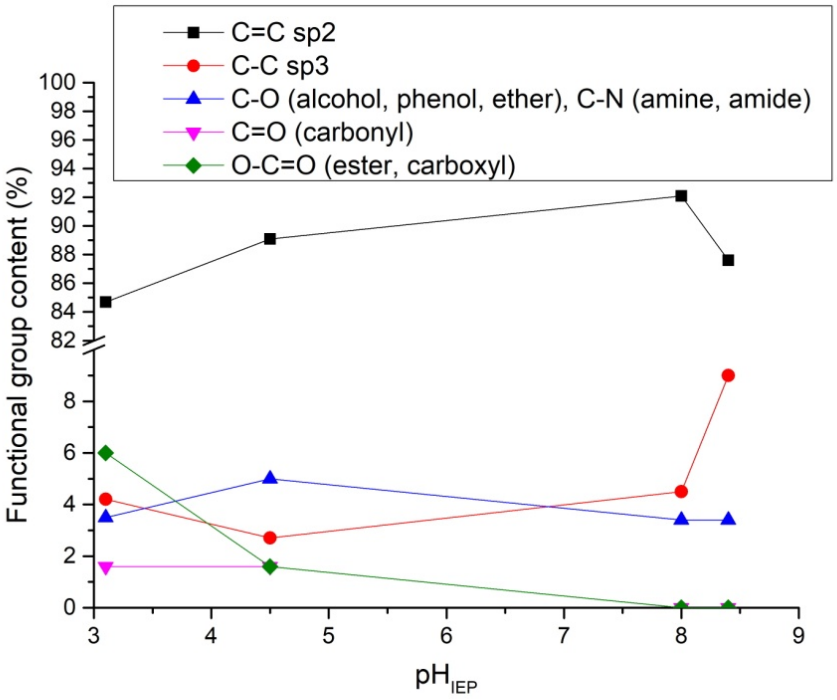

Figure S9. Functional group content versus pH_{IEP} of the carbons studied.

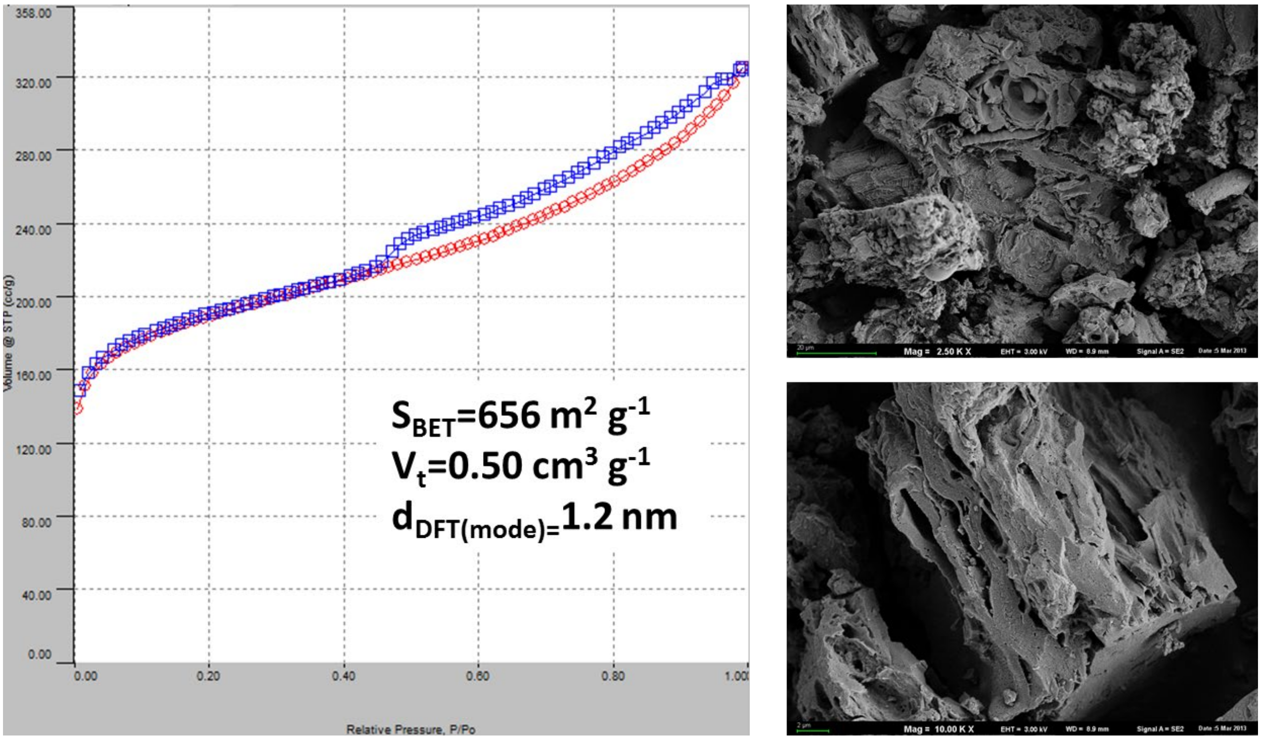

Figure S10. Nitrogen adsorption isotherm of Norit SX2 (left), SEM images of Norit SX2 (right).

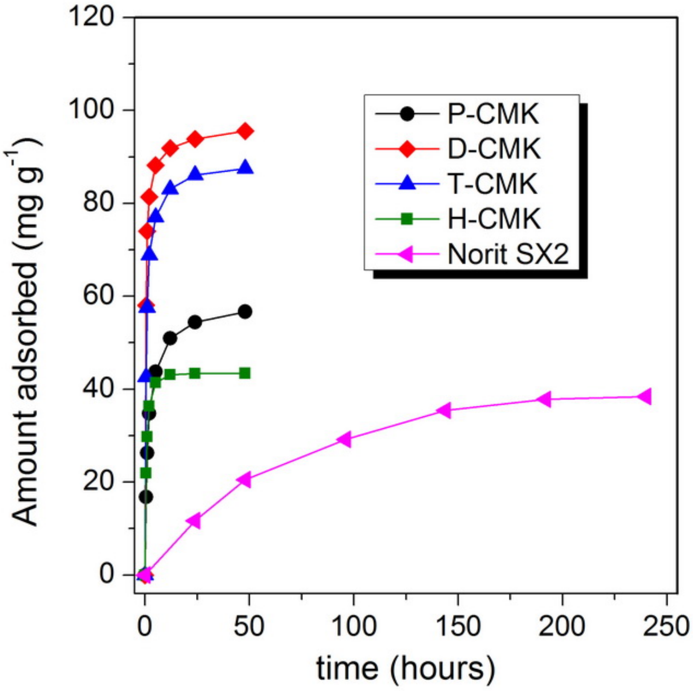

Figure S11. Comparison of DICL adsorption kinetics onto the studied CMK materials and Norit SX2 carbon (initial concentration of DICL: 50 mg L^{-1}).

Table S1. Results of the deconvolution of the XPS C 1s and O 1s core energy levels.

Binding Energy (eV)	Bond Assignment	P-CMK	D-CMK	T-CMK	H-CMK
	C 1s	91.0	94.8	92.4	81.8
283.9-284.2	C=C sp^2	90.6	90.4	85.7	84.8
284.8-285.6	C–C sp^3	2.8	4.4	8.8	4.1
285.9-286.1	C–O (alcohol, phenol, ether), C–N (amine, amide)	5.0	3.4	3.4	3.5
286.6-286.8	C=O (carbonyl)	1.6	-	-	1.6
288.2-288.6	O–C=O (ester, carboxyl)	-	1.8	2.1	6.0
	O 1s	7.0	5.2	4.5	15.9
530.8-531.0	O=C (carbonyl)	21.7	2.6	5.1	11.2
531.4-531.6	O^*=C–O (ester, carboxyl)	21.7	-	-	33.1
532.2-532.6	Aliphatic C–O (alcohol, phenol)	36.8	44.9	53.1	25.4
533.5-533.8	Aromatic C–O (ether)	19.8	52.5	41.8	30.3

materials

MDPI

Table S2. Comparison of DICL maximum adsorption capacities by carbon-derived sorbents reported in the literature.

Sorbent	Observed Uptake ($mg\ g^{-1}$)	Remarks	Ref.
Oxidized activated carbon (treated with a solution of ammonium persulfate and sulfuric acid)	487 $mg\ g^{-1}$	Optimal pH: 5.5–6.0. Oxidation increases adsorbed amount 6 times. Proposed mechanism based on electrostatic interactions and hydrogen bonding. Desorption by acetone provides up to 5 reusable cycles.	[1]
Multi-walled carbon nanotubes treated with dilute nitric acid	24 $mg\ g^{-1}$	Opt. pH: 5.0. Fast ($t_{eq} \approx 1$ h) and multilayered adsorption was observed.	[2]
Graphene oxide reduced by sodium borohydride	60 $mg\ g^{-1}$	Opt. pH: 10.0. Adsorption equilibrium reached after 3 h. Proposed mechanism based on π–π interactions, electrostatic attraction and hydrogen bonding.	[3]
Activated carbon from cocoa shell	64 $mg\ g^{-1}$	Opt. pH: 7.0, $t_{eq} \approx 4$ h. Proposed mechanism based on π–π−stacking, hydrogen bonding and van der Waals forces. AC effectively removed 96% of a mixture of different organic compounds in a medium with high salinity and sugar content.	[4]
Activated carbon from agricultural by-product	56 $mg\ g^{-1}$	Opt. pH: 7.0, $t_{eq} > 5$ h. Proposed mechanism based on π–π stacking, hydrogen bonding and/or van der Waals forces.	[5]
Graphene oxide	500 $mg\ g^{-1}$	Opt. pH: 7.0, $t_{eq} \approx 24$ h. Proposed mechanism based on hydrophobic interactions and π–π stacking.	[6]
Expanded graphite	330 $mg\ g^{-1}$	Fast (Eq. time≈ 0.5 h) adsorption onto energetically uniform carbon surface.	[7]
Activated carbon from olive stones	11 $mg\ g^{-1}$	Opt. pH: 2.0. Fast ($t_{eq} \approx 0.5$ h) adsorption of DICL related to film diffusion and intraparticle diffusion.	[8]
Activated carbon from *Terminalia catappa*	91 $mg\ g^{-1}$	Opt. pH: 5.0; $t_{eq} \approx 2$ h. Proposed mechanism based on hydrogen bonding. Desorption at pH = 5 and 60 °C provides up to 8 reuses with 85% removal.	[9]
Carbon derived from TiC by chlorination	551 $mg\ g^{-1}$	Fast ($t_{eq} \approx 0.5$ h), selective and multilayered adsorption was observed.	[10]
Activated carbon cloth	414 $mg\ g^{-1}$	Opt. pH: 7.5; $t_{eq} > 20$ days. Oxidation decreases adsorption capacity. Proposed mechanism based on dispersive and hydrophobic interactions.	[11]
Activated carbon, multi-walled carbon nanotubes and carbon nanofibers	329 $mg\ g^{-1}$	Slow ($t_{eq} > 14$ days) and non-selective adsorption was observed.	[12]
Iron-enriched magnetic biocarbon	316 $mg\ g^{-1}$	Opt. pH: 5; $t_{eq} > 3$ h. Proposed mechanism based on electrostatic interactions, hydrogen bonding and π–π stacking. Desorption by acetone provides up to 4 recyclable runs.	[13]
Iron-enriched activated carbon from orange peels	144 $mg\ g^{-1}$	Opt. pH:4.5; $t_{eq} > 3$ h. Proposed mechanism based on hydrogen bonding, π–π stacking, ion-dipole interactions and Fenton-like degradation.	[14]
Hydrochar from dried fruit powder	601 $mg\ g^{-1}$	Opt. pH: 4.4. Fast ($t_{eq} \approx 1.5$ h) and physical adsorption was observed.	[15]
CO_2-activated carbon from coconut shell	1033 $mg\ g^{-1}$	Opt. pH: 7.0; $t_{eq} > 7$ days. Proposed mechanism based on π–π stacking and electrostatic interactions.	[16]
3D reduced graphene oxide aerogel	597 $mg\ g^{-1}$	Opt. pH: 6.0, $t_{eq} \approx 1$ h. Proposed mechanism based on electrostatic attraction, π–π stacking, hydrogen bonding and hydrophobic interactions.	[17]
Activated carbon from tea waste	62 $mg\ g^{-1}$	Opt. pH: 6.5; $t_{eq} > 6$ h. Spontaneous, endothermic and physical adsorption was observed.	[18]
Multi-walled carbon nanotubes	6 $mg\ g^{-1}$	Opt. pH: 7.0; $t_{eq} \approx 0.5$ h. Desorption by 0.1 M HCl provides 1 reuse cycle.	[19]
Thermochemically modified CMK-3 carbon	241 $mg\ g^{-1}$	Opt. pH ≈ 5.5–6.0. Fast adsorption kinetics, possibility of partial regeneration	This work

References

1. Bhadra, B.N.; Seo, P.W.; Jhung, S.H. Adsorption of diclofenac sodium from water using oxidized activated carbon. *Chem. Eng. J.* **2016**, *301*, 27–34.
2. Hu, X.; Cheng, Z. Removal of diclofenac from aqueous solution with multi-walled carbon nanotubes modified by nitric acid. *Chinese J. Chem. Eng.* **2015**, *23*, 1551–1556.
3. Jauris, I.M.; Matos, C.F.; Saucier, C.; Lima, E.C.; Zarbin, A.J.G.; Fagan, S.B.; Machado, F.M.; Zanella, I. Adsorption of sodium diclofenac on graphene: a combined experimental and theoretical study. *Phys. Chem. Chem. Phys.* **2016**, *18*, 1526–1536.
4. Saucier, C.; Adebayo, M.A.; Lima, E.C.; Cataluña, R.; Thue, P.S.; Prola, L.D.T.; Puchana-Rosero, M.J.; Machado, F.M.; Pavan, F.A.; Dotto, G.L. Microwave-assisted activated carbon from cocoa shell as adsorbent for removal of sodium diclofenac and nimesulide from aqueous effluents. *J. Hazard. Mater.* **2015**, *289*, 18–27.
5. Baccar, R.; Sarrà, M.; Bouzid, J.; Feki, M.; Blánquez, P.; Sarr??, M.; Bouzid, J.; Feki, M.; Bl??nquez, P. Removal of pharmaceutical compounds by activated carbon prepared from agricultural by-product. *Chem. Eng. J.* **2012**, *211–212*, 310–317.
6. Nam, S.W.; Jung, C.; Li, H.; Yu, M.; Flora, J.R. V; Boateng, L.K.; Her, N.; Zoh, K.D.; Yoon, Y. Adsorption characteristics of diclofenac and sulfamethoxazole to graphene oxide in aqueous solution. *Chemosphere* **2015**, *136*, 20–26.
7. Vedenyapina, M.D.; Borisova, D.A.; Simakova, A.P.; Proshina, L.P.; Vedenyapin, A.A. Adsorption of diclofenac sodium from aqueous solutions on expanded graphite. *Solid Fuel Chem.* **2013**, *47*, 59–63.
8. Larous, S.; Meniai, A.-H. Adsorption of Diclofenac from aqueous solution using activated carbon prepared from olive stones. *Int. J. Hydrogen Energy* **2016**, *41*, 10380–10390.
9. Sathishkumar, P.; Arulkumar, M.; Ashokkumar, V.; Mohd Yusoff, A.R.; Murugesan, K.; Palvannan, T.; Salam, Z.; Ani, F.N.; Hadibarata, T. Modified phyto-waste Terminalia catappa fruit shells: a reusable adsorbent for the removal of micropollutant diclofenac. *RSC Adv.* **2015**, *5*, 30950–30962.
10. Álvarez-Torrellas, S.; Munoz, M.; Gläsel, J.; de Pedro, Z.M.; Domínguez, C.M.; García, J.; Etzold, B.J.M.; Casas, J.A. Highly efficient removal of pharmaceuticals from water by well-defined carbide-derived carbons. *Chem. Eng. J.* **2018**, *347*, 595–606.
11. Masson, S.; Gineys, M.; Delpeux-Ouldriane, S.; Reinert, L.; Guittonneau, S.; Béguin, F.; Duclaux, L. Single, binary, and mixture adsorption of nine organic contaminants onto a microporous and a microporous/mesoporous activated carbon cloth. *Microporous Mesoporous Mater.* **2016**, *234*, 24–34.
12. Sotelo, J.L.; Rodríguez, A.R.; Mateos, M.M.; Hernández, S.D.; Torrellas, S.A.; Rodríguez, J.G. Adsorption of pharmaceutical compounds and an endocrine disruptor from aqueous solutions by carbon materials. *J. Environ. Sci. Heal. Part B* **2012**, *47*, 640–652.
13. Luo, H.; Zhang, Y.; Xie, Y.; Li, Y.; Qi, M.; Ma, R.; Yang, S.; Wang, Y. Iron-rich microorganism-enabled synthesis of magnetic biocarbon for efficient adsorption of diclofenac from aqueous solution. *Bioresour. Technol.* **2019**, *282*, 310–317.
14. Tomul, F.; Arslan, Y.; Başoğlu, F.T.; Babuçcuoğlu, Y.; Tran, H.N. Efficient removal of anti-inflammatory from solution by Fe-containing activated carbon: Adsorption kinetics, isotherms, and thermodynamics. *J. Environ. Manage.* **2019**, *238*, 296–306.
15. Qureshi, T.; Memon, N.; Memon, S.Q.; Yavuz, H.; Lachgar, A.; Denizli, A. Evaluation of hydrochar efficiency for simultaneous removal of diclofenac and ibuprofen from aqueous system using surface response methodology. *Environ. Sci. Pollut. Res.* **2019**, *26*, 9796–9804.
16. Moral-Rodríguez, A.I.; Leyva-Ramos, R.; Ania, C.O.; Ocampo-Pérez, R.; Isaacs-Páez, E.D.; Carrales-Alvarado, D.H.; Parra, J.B. Tailoring the textural properties of an activated carbon for enhancing its adsorption capacity towards diclofenac from aqueous solution. *Environ. Sci. Pollut. Res.* **2019**, 1–12.
17. Hiew, B.Y.Z.; Lee, L.Y.; Lai, K.C.; Gan, S.; Thangalazhy-Gopakumar, S.; Pan, G.-T.; Yang, T.C.-K. Adsorptive decontamination of diclofenac by three-dimensional graphene-based adsorbent: Response surface methodology, adsorption equilibrium, kinetic and thermodynamic studies. *Environ. Res.* **2019**, *168*, 241–253.

18. Malhotra, M.; Suresh, S.; Garg, A. Tea waste derived activated carbon for the adsorption of sodium diclofenac from wastewater: adsorbent characteristics, adsorption isotherms, kinetics, and thermodynamics. *Environ. Sci. Pollut. Res.* **2018**, *25*, 32210–32220.
19. Gil, A.; Santamaría, L.; Korili, S.A. Removal of Caffeine and Diclofenac from Aqueous Solution by Adsorption on Multiwalled Carbon Nanotubes. *Colloid Interface Sci. Commun.* **2018**, *22*, 25–28.

© 2020 by the authors. Submitted for possible open access publication under the terms and conditions of the Creative Commons Attribution (CC BY) license (http://creativecommons.org/licenses/by/4.0/).
